# Can we cure stage IV triple-negative breast carcinoma? Another case report of long-term survival (7 years)

**DOI:** 10.1097/MD.0000000000017251

**Published:** 2019-09-20

**Authors:** Ben Man-Fai Chue, Bryce Douglas La Course

**Affiliations:** Lifespring Cancer Treatment Center, Seattle, WA.

**Keywords:** immunotherapy, long-term survival, metastatic triple-negative breast cancer, metronomic chemotherapy, sequential chemotherapy regimens

## Abstract

**Rationale:**

Triple-negative breast cancer has a dismal prognosis, especially once it has spread to other organs, due to the lack of effective treatments available at this time. Finding an effective treatment for metastatic triple-negative breast cancer remains an unmet medical need.

**Patient concerns:**

A 60-year-old woman was diagnosed with stage IIIC triple-negative breast cancer after undergoing a mastectomy. Her mastectomy was followed by adjuvant chemotherapy and radiation therapy. Approximately 1 year later, the patient presented with enlarging lymph nodes in her neck. A biopsy of a left supraclavicular lymph node was positive for recurrent disease. Positron emission tomography and computed tomography scans performed after the biopsy showed metabolic activity in the T6 vertebral body and the right level IIB lymph nodes.

**Diagnoses:**

The patient was diagnosed with recurrent metastatic triple-negative breast carcinoma with metastases to the bone and lymph nodes.

**Interventions:**

The patient was treated with weekly metronomic chemotherapy, sequential chemotherapy regimens, and immunotherapy.

**Outcomes:**

The patient is now 68 years old and 7 years out from her diagnosis of metastatic disease. She achieved a complete response to her treatment and routine scans continue to show no evidence of recurrent disease.

**Lessons:**

Utilizing sequential weekly metronomic chemotherapy regimens in combination with immunotherapy looks to be a promising treatment option for patients with metastatic triple-negative breast carcinoma. This is a second case where we were able to achieve long-term remission by using the above treatment strategy. These exciting results warrant further investigation of this treatment methodology. We hope that the treatment strategy described in this article can provide an outline for researchers and give patients with this disease more treatment options.

## 1 Introduction

Breast cancer accounts for 30% of all new cancer diagnoses in women and remains the second leading cause of cancer-related deaths in the United States. Approximately 42,000 patients are projected to die from breast cancer in 2019 alone.^[1]^ Breast cancers that do not express the estrogen receptor (ER), progesterone receptor (PR) or overexpress human epidermal growth factor receptor 2 (HER2) are commonly referred to as “triple-negative breast cancer (TNBC)” and represent approximately 11% of all breast cancers.^[2]^ This subtype of breast cancer has a dismal prognosis, especially once the disease has metastasized. Even with treatment, the median overall survival of patients with metastatic TNBC is only about 13 months.^[3]^ Currently, palliative chemotherapy is the standard of care for patients with metastatic disease, but unfortunately, most patients progress quickly on treatment. Due to its poor prognosis and lack of effective treatments, there is a need for the development of novel treatment strategies that can improve the survival of patients with metastatic TNBC. Previously, we reported a case of a patient with metastatic TNBC who is now over 15 and a half years out from her diagnosis of metastatic disease and remains in complete remission.^[4]^ Here, we report the second case of a patient with metastatic TNBC who is currently 7 years out from her diagnosis of metastatic disease without any evidence of persistent or recurrent cancer. This raises the possibility of a cure for this disease.

## 2 Case report

A Caucasian female initially presented with nodules in her right breast in early June 2011 at the age of 60. The patient previously worked as a recruiter and had a history of type 2 diabetes mellitus. Her mother deceased at age 75 and her maternal aunt deceased at age 72, both having a history of lung cancer. The patient socially drank alcohol but did not use tobacco. She underwent a mammogram which showed an abnormal mass. A subsequent positron emission tomography (PET) and computed tomography (CT) scan confirmed the presence of the right breast mass with possible lymph node metastases, but there were no other areas of disease at the time. A biopsy of the breast mass was performed which showed an invasive ductal carcinoma that was negative for ER and PR expression as well as HER2 overexpression. The patient underwent a right modified radical mastectomy in late June 2011. Surgical pathology showed 19 lymph nodes were positive for metastatic disease. The patient proceeded with adjuvant chemotherapy and completed 6 cycles of docetaxel, doxorubicin, and cyclophosphamide in November 2011. A repeat PET/CT scan was negative for any residual or metastatic disease. She then underwent adjuvant radiation therapy to the right breast and axilla and completed treatment in January 2012. A PET/CT scan done in February 2012 was negative for any recurrent disease.

**Figure 1:**
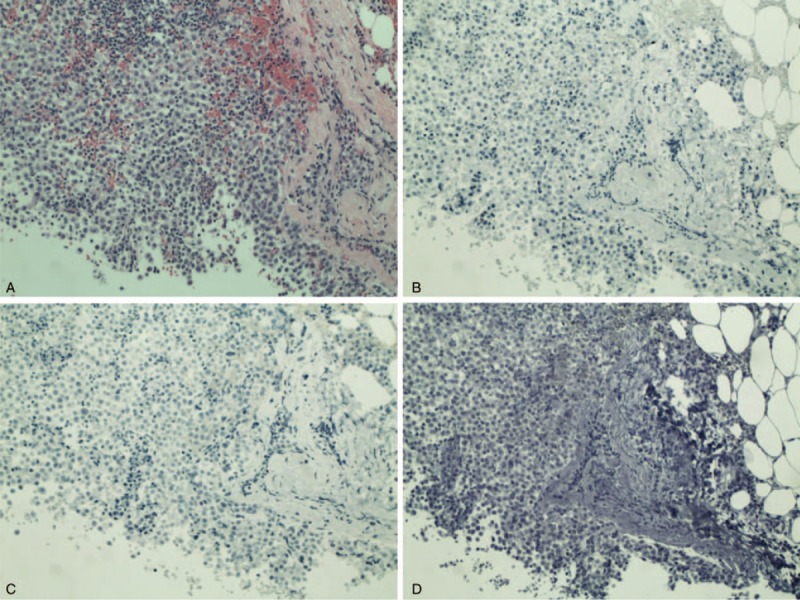
Analysis of the biopsy specimen of the left supraclavicular lymph node from June 2012 revealed a metastatic carcinoma seen with hematoxylin and eosin staining (A). Immunohistochemical staining showed that the tumor was negative for estrogen receptor expression (B), negative for progesterone receptor expression (C), and negative for human epidermal growth factor receptor 2 overexpression (D).

**Figure 2:**
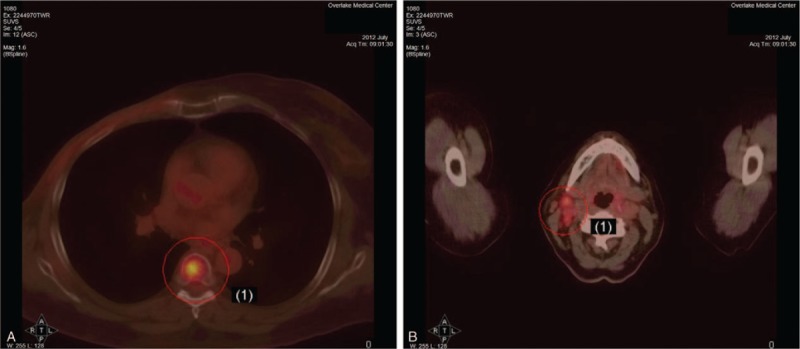
Positron emission tomography and computed tomography axial fused images from July 2012 showed a T6 vertebral body osteolytic defect which measured 1.6 cm and had a maximum sugar uptake value of 5.5 (A). There were also 2 metabolically active enlarged right level IIB lymph nodes. The largest lymph node at the angle of the mandible measured 1.7 × 1.4 cm and the second bilobed lymph node measured 1.9 × 1.1 cm. This cluster of lymph nodes had a maximum sugar uptake value of 4.0 (B).

In June 2012, the patient noticed an enlarging left supraclavicular lymph node. An ultrasound-guided core biopsy revealed a recurrent metastatic breast carcinoma that was morphologically similar to the previously diagnosed tumor in the right breast. The tumor was again negative for ER, PR, and HER2 by immunohistochemistry (Fig. 1). These studies were repeated and reviewed by a second pathologist who confirmed the findings. A PET/CT scan performed in July 2012 showed metabolic activity in a new scalloped osteolytic defect of the T6 vertebral body and enlarged right level IIB lymph nodes that were consistent with metastases (Fig. 2). The biopsy-proven metastasis to the left supraclavicular lymph node had minimal metabolic activity. The patient was diagnosed as having recurrent metastatic TNBC with metastases to the bone and lymph nodes. The patient started on palliative chemoradiation in mid-July 2012 but decided to discontinue chemoradiation a day later.

The patient then came to our clinic for a second opinion. We recommended systemic chemotherapy treatment alone and she decided to proceed with our treatment. She received 7 chemotherapy regimens that were administered on a weekly basis from late July 2012 to June 2014 (Table 1) to control her cancer. She also received monthly zoledronic acid to try to prevent bone complications due to her osseous metastasis. Granulocyte-macrophage colony-stimulating factor (GM-CSF) was used throughout the treatment to prevent or treat chemotherapy-induced neutropenia as well as stimulate the immune system.

**Table 1:**
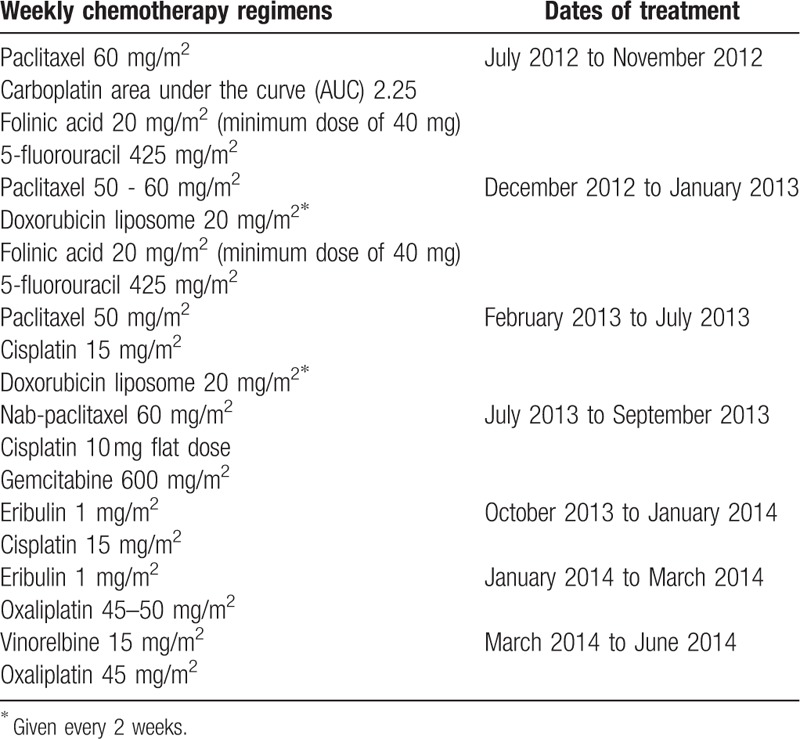
Treatment history and dosing.

**Figure 3:**
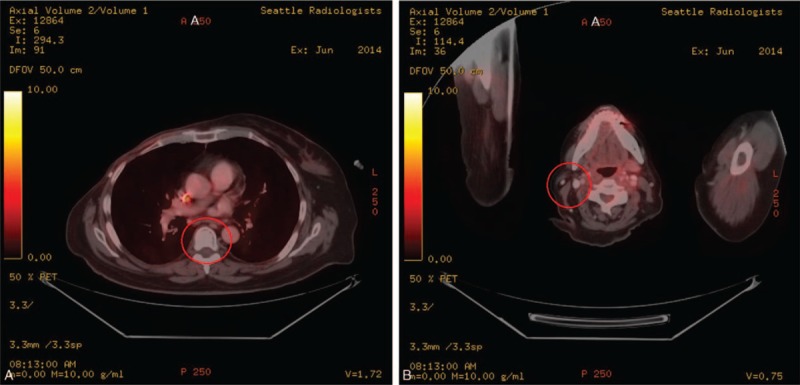
Positron emission tomography and computed tomography axial fused images from June 2014 showed interval sclerosis of the lesion within the T6 vertebra without focal radiotracer accumulation within this bone lesion. There was some focal radiotracer accumulation within the distal tip of the left portacatheter within the superior vena cava which was noted to be due to a small thrombus (A). The previous metabolically active right level IIB lymphadenopathy had resolved (B).

The patient's first chemotherapy regimen consisted of weekly paclitaxel and carboplatin. Folinic acid and 5-fluorouracil (5-FU) were added to the patient's treatment regimen during her second dose to increase treatment efficacy. A CT scan performed in November 2012 after 12 doses of the carboplatin-based regimen showed interval sclerosis of the T6 vertebral body metastasis and stability in the size of her left supraclavicular lymph node. Her treatment was then switched to an anthracycline-based regimen consisting of paclitaxel, doxorubicin liposome, folinic acid, and 5-FU. Doxorubicin liposome was given every 2 weeks, but paclitaxel, folinic acid, and 5-FU remained on a weekly schedule. After receiving 6 doses of this regimen, folinic acid and 5-FU were discontinued due to the development of grade 2 oral mucositis. Folinic acid and 5-FU were replaced with low-dose cisplatin. She received 16 doses of her new anthracycline-based regimen that consisted of paclitaxel, doxorubicin liposome, and low-dose cisplatin. After her 11th dose of this regimen, paclitaxel was switched to nab-paclitaxel for better tolerance. She was also started on low-dose interferon-alpha-2b (IFN) for its immunostimulatory effects at this time. A PET/CT scan performed in July 2013 demonstrated a densely sclerotic lesion in the T6 vertebral body without metabolic activity and no lymphadenopathy, consistent with a good disease response to treatment. The patient was then switched to a gemcitabine-based regimen consisting of nab-paclitaxel, low-dose cisplatin, and gemcitabine. She received 4 doses of this regimen but developed grade 3 thrombocytopenia secondary to gemcitabine. Due to the concerns of inconsistent treatment compromising efficacy, she was switched to an eribulin-based regimen that consisted of eribulin and low-dose cisplatin. The patient received 12 doses of this eribulin-based regimen which was tolerated much better. Her regimen was then switched to another eribulin-based regimen consisting of eribulin and oxaliplatin. She received 3 doses of this regimen and then eribulin was discontinued due to developing grade 2 thrombocytopenia and grade 3 anemia. The patient then continued on a vinorelbine-based regimen that consisted of vinorelbine and oxaliplatin. After the 4th dose of this regimen, oxaliplatin was discontinued due to her cytopenias. She then received another 5 doses of vinorelbine alone. The patient finished chemotherapy treatment and discontinued low-dose IFN in June 2014. Her blood counts dropped more than expected while on chemotherapy treatment, but this was thought to be due to the bone involvement of her cancer and her history of radiation therapy. The patient underwent a repeat PET/CT scan in early June 2014 after completing chemotherapy treatment which showed a stable T6 sclerotic bone lesion without any metabolic activity and no other evidence of metastatic disease, an overall complete response as defined by the response evaluation criteria in solid tumors (Fig. 3). In January 2015, the patient discontinued zoledronic acid due to the development of grade 2 osteonecrosis of the jaw.

Routine CT scans for surveillance have continued to demonstrate no evidence of persistent or recurrent metastatic disease. The patient is now 68 years old and continues to enjoy a relatively good quality of life. She is currently 7 years out from her diagnosis of recurrent metastatic disease and has not received chemotherapy treatment since June 2014.

## 3 Discussion

The typical prognosis of patients with metastatic TNBC is poor, with most patients only surviving slightly over 1 year with treatment, making this patient quite remarkable since she is currently 7 years out from her diagnosis of metastatic disease. This patient's long-term survival is likely due to the treatment she received, which included weekly metronomic chemotherapy, sequential chemotherapy regimens, and immunotherapy. We previously reported treating another patient with metastatic TNBC using a similar treatment strategy who is now over 15 and a half years out from her diagnosis of metastatic disease without any evidence of recurrence.^[4]^ This would make the patient described here our second remarkable case. We have also treated metastatic pancreatic cancer using this treatment strategy and have achieved long-term survival.^[5–8]^ We will describe each part of this patient's treatment below.

### 3.1 Metronomic chemotherapy

Most chemotherapy regimens are administered every 3 weeks with dosing based on a drug's maximum tolerated dose, which is the highest dose of a drug that is medically feasible. This idea was originally developed with the logic that a higher dose of chemotherapy will maximize the amount of cancer cell death. Standard chemotherapy regimens have been highly effective for the treatment of certain types of cancer, such as testicular cancer and Hodgkin's lymphoma, but have not been as successful for most other cancers.^[9]^ In addition, utilizing a standard dose of chemotherapy is well known for causing toxic side effects that can decrease a patient's quality of life and interrupt regularly scheduled treatments.

Instead of receiving a high dose of chemotherapy every 3 weeks, this patient received lower doses of chemotherapy on a weekly basis. The idea of administering lower doses of cytotoxic drugs more frequently without drug-free breaks is commonly called “metronomic chemotherapy.” More evidence is accumulating which strongly suggests that certain combinations of chemotherapy agents that are administered metronomically can be at least as effective, if not more effective, when compared to standard treatment.^[10–12]^ The increased treatment efficacy of metronomic chemotherapy regimens can be explained by their increased dose density and dose intensity as well as the many effects that this dosing schedule has on the tumor microenvironment. One of the most well-studied effects of metronomic chemotherapy has been its anti-angiogenic effects.^[13]^ There are also some studies showing that metronomic chemotherapy with certain agents can also have anti-stromal and immunostimulatory effects.^[14–16]^ In addition to potentially being more effective, this dosing schedule typically has a favorable toxicity profile because a lower dose of chemotherapy is administered during each treatment, allowing for better quality of life.^[17]^

### 3.2 Sequential chemotherapy regimens

Currently, it is common for a single chemotherapy regimen to be continued until there is obvious disease progression. This idea was likely developed with the logic of trying to maximize the window of therapeutic benefit of a specific chemotherapy regimen. However, this treatment norm will also inadvertently select for cancer cells that have developed ways to escape the cytotoxic effects of a specific treatment, ultimately resulting in a new population of cancer cells that can resist treatment.^[18]^ Switching a chemotherapy regimen before noticeable disease progression may be able to prevent the development of chemotherapy resistance, especially if the next regimen contains chemotherapy agents with a different mechanism of action. In addition, switching treatments can prevent the accumulation of regimen-specific side effects, which might also be able to improve a patient's quality of life during treatment. The idea of sequential chemotherapy regimens is not new and has found success in the treatment of metastatic non-small cell lung carcinoma and pediatric acute lymphoblastic leukemia.^[19,20]^

By switching chemotherapy regimens before disease progression, we believe we were able to gradually decrease the number of cancer cells over time to a very low level without losing progress due to disease progression in between regimens. Since the patient had already received adjuvant chemotherapy treatment, there was a concern that her recurrent metastatic disease harbored some degree of chemotherapy resistance which is why she continued on chemotherapy treatment even when a PET/CT scan performed a year into her treatment showed no lymphadenopathy and a sclerotic lesion in the T6 vertebral body without metabolic activity.

To increase treatment efficacy due to concerns of potential chemotherapy resistance, 5-FU was added to this patient's carboplatin-based and doxorubicin liposome-based regimens. 5-FU has been shown to be effective in combination with platinum chemotherapy agents and has been paired with anthracyclines in the 5-FU, epirubicin, and cyclophosphamide and 5-FU, doxorubicin, and cyclophosphamide chemotherapy regimens for the treatment of breast cancer.^[21]^ Capecitabine, an analog of 5-FU, is also commonly used for the treatment of metastatic TNBC. The addition of 5-FU to this patient's regimen of paclitaxel and carboplatin was tolerated without any issues, but she developed mucositis when 5-FU was combined with paclitaxel and doxorubicin liposome. The combination of paclitaxel, doxorubicin liposome, and low-dose cisplatin was tolerated much better and is what we have administered to subsequent patients. Instead of adding a platinum agent to eribulin as this patient received, 5-FU could be added instead to make treatment more tolerable, which is also what we have done for other patients. It may be important to incorporate 5-FU into several chemotherapy regimens not only because 5-FU might increase treatment efficacy, but also because 5-FU can cross the blood–brain barrier and potentially aid in the treatment of metastatic TNBC that has spread to the central nervous system. In addition, platinum chemotherapy agents were used in the majority of the patient's chemotherapy regimens because platinum chemotherapy agents have been shown to improve overall survival in patients with metastatic TNBC.^[22]^

In our experience, the 2 patients who achieved long-term disease-free survival with metastatic TNBC both received a carboplatin-based regimen, followed by a doxorubicin liposome-based regimen, a gemcitabine-based regimen, and then a vinorelbine-based regimen. The logic behind using these regimens was previously discussed in detail.^[4]^ Other treatments, such as an eribulin-based regimen, may also be effective as shown in this patient's case. The optimum combination of chemotherapy agents used in each regimen and the order of chemotherapy regimens will need to be investigated further. In addition, the ideal length of time before chemotherapy regimens are switched will need to be determined, but we have had success switching treatments after about 12 weekly doses.

### 3.3 Immunotherapy

Although there are currently no targeted therapies approved specifically for the treatment of metastatic TNBC, this type of cancer appears to be relatively susceptible to immunotherapies.^[23]^ In addition to metronomic chemotherapy, which is thought to have immunomodulatory properties, this patient also received several other immunotherapies.

While receiving treatment, the patient received 250 to 500 mcg of GM-CSF up to 5 days each week to prevent or treat chemotherapy-induced neutropenia. The other long-term survivor we reported on also received GM-CSF with her treatment.^[4]^ In addition to increasing a patient's neutrophil count, GM-CSF is thought to have immunostimulatory properties due to the stimulation of monocyte/macrophage and dendritic cell lines which are necessary for an adaptive immune response.^[24]^ Typically, GM-CSF is administered subcutaneously, but a small amount of GM-CSF was first administered intradermally and then the remaining medication was administered subcutaneously due to the abundance of immune cells in the intradermal layer.^[25]^

About halfway through her treatment, the patient also began administering 300,000 international units of IFN intradermally 3 times per week. IFN has known anti-tumor activity in several types of cancer and is thought to work by stimulating natural killer cells, macrophages, and helper T cells, as well as by having direct antiproliferative effects.^[26]^ At standard doses, IFN can be difficult to tolerate, but the low dose of IFN this patient received was tolerated without any issues. Normally, IFN is administered subcutaneously or intramuscularly, but the intradermal route was chosen for the same reason we administered GM-CSF intradermally. During treatment, GM-CSF and low-dose IFN may have synergistic antitumor effects, especially when used in conjunction with metronomic chemotherapy treatments which are thought to have immunomodulatory properties. Using low-dose IFN in a patient's treatment may also play a role in achieving long-term survival.^[6]^

### 3.4 Potential Implications

In our previous report, we suggested that there may be significant economic implications of using this treatment strategy.^[4]^ The growing cost of cancer care in the United States is not sustainable and breast cancer is projected to have one of the largest increases in cost overall.^[27]^ Immune checkpoint inhibitors and poly adenosine diphosphate ribose polymerase inhibitors are exciting new treatments for metastatic TNBC, but at the present time only seem to benefit specific subgroups of these patients and at a substantial cost.^[28,29]^ By continuing to use expensive drugs, this not only impacts a patient's ability to afford the treatment they require, but also our healthcare system as a whole.

One study suggests that metronomic chemotherapy can reduce healthcare costs when compared to other novel chemotherapy strategies, although this may vary depending on the chemotherapy regimen being used.^[30]^ The metronomic dosing of chemotherapy, as well as the use of sequential chemotherapy regimens and the immunotherapies outlined above, may be able to decrease healthcare costs because this treatment strategy uses mostly generic chemotherapy agents, the immunotherapies used are relatively inexpensive, and there are less severe side effects which could lead to fewer emergency room visits and hospitalizations. Furthermore, if patients can completely stop their cancer treatment after a period of time, just as this patient and the patient we previously described have done, this could result in less healthcare expenditure overall. The economic benefits and cost-effectiveness of this treatment strategy should be investigated further.

## 4 Conclusion

This report describes a second case of long-term sustained complete remission with metastatic TNBC. This patient is 7 years out from her diagnosis of recurrent metastatic disease, a diagnosis that usually has a prognosis of about 1 year. We believe this patient's long-term disease-free survival is likely due to the treatment she received which included sequential weekly metronomic chemotherapy regimens as well as several immunotherapies. This raises the question of the possibility of a cure for metastatic TNBC as well as other metastatic cancers. We hope that these exciting results are investigated further and confirmed either by controlled studies or by the accumulation of real-world data because of the substantial medical, psychological, and economic implications. Furthermore, it might be worthwhile to see if some of these regimens are also effective in the neoadjuvant and adjuvant settings for patients with earlier-stage disease, especially the carboplatin-based and doxorubicin liposome-based regimens described here.

## Acknowledgments

The authors would like to thank Dr. Hongxiu Ji, MD, for her help in providing pathology slide images, and Zhu Yesu, without whom none of this work could have been done.

## Author contributions

**Conceptualization:** Ben Man-Fai Chue, Bryce Douglas La Course.

**Formal analysis:** Ben Man-Fai Chue, Bryce Douglas La Course.

**Writing – original draft:** Ben Man-Fai Chue, Bryce Douglas La Course.

**Writing – review & editing:** Ben Man-Fai Chue, Bryce Douglas La Course.
